# Supplementary material for: Genomic evidence for a-α heterothallic and α-α unisexual mating and recombination in an environmental Cryptococcus deneoformans population
Source: PLoS Genet. 2025 Dec 4;21(12):e1011844. doi: 10.1371/journal.pgen.1011844 (PMC12677459; doi:10.1371/journal.pgen.1011844)
Supplement: S1 File — One criterion used 60% of bases over Q30 and the other used 85% of bases over Q30. Only SNPs with rare allele count of at least two in the population, SNPs that were analyzed in this study, are shown in this file. (DOCX) [file pgen.1011844.s001.docx]

S1 File. Distribution of biallelic SNPs with the rare allele count being at least two in the total population of 24 isolates.

| Chromosome | Total population SNPs in Manuscript  (60% bases over Q30) | Total Population with stricter filtering (85% bases over Q30) |
| --- | --- | --- |
| 1 | 13512 | 13506 |
| 2 | 9765 | 9755 |
| 3 | 10769 | 10771 |
| 4 | 9761 | 9753 |
| 5 | 8431 | 8427 |
| 6 | 7607 | 7618 |
| 7 | 7575 | 7582 |
| 8 | 6499 | 6485 |
| 9 | 6602 | 6601 |
| 10 | 4700 | 4718 |
| 11 | 6407 | 6405 |
| 12 | 4420 | 4414 |
| 13 | 4259 | 4251 |
| 14 | 4486 | 4485 |
| Total nuclear genome | 104793 | 104771 |
